# Outcomes, Mortality Causes, and Pathological Findings in European Hedgehogs (*Erinaceus europeus*, Linnaeus 1758): A Seventeen Year Retrospective Analysis in the North of Portugal

**DOI:** 10.3390/ani10081305

**Published:** 2020-07-30

**Authors:** Andreia Garcês, Vanessa Soeiro, Sara Lóio, Roberto Sargo, Luís Sousa, Filipe Silva, Isabel Pires

**Affiliations:** 1 Inno–Serviços Especializados em Veterinária, R. Cândido de Sousa 15, 4710-300 Braga, Portugal; 2 Wildlife Rehabilitation Centre of Parque Biológico de Gaia, Rua da Cunha, 4430-812 Avintes, Portugal; vanessasoeiro@cm-gaia.pt (V.S.); saraloio@cm-gaia.pt (S.L.); 3 Wildlife Rehabilitation Centre of University of Trás-os-Montes and Alto Douro (CRAS-UTAD), University of Trás-os-Montes and Alto Douro, Quinta de Prados, 5000-801 Vila Real, Portugal; rsargo@utad.pt (R.S.); luissousa@utad.pt (L.S.); fsilva@utad.pt (F.S.); 4 CECAV, University of Trás-os-Montes and Alto Douro, Quinta de Prados, 5000-801 Vila Real, Portugal; ipires@utad.pt

**Keywords:** *Erinaceus europaeus*, hedgehog, Portugal, mortality, trauma, pathology

## Abstract

**Simple Summary:**

The Western European hedgehog *Erinaceus europaeus* (Linnaeus, 1758) is one of the most common mammals in urban areas. We collected data over 17 years (2002–2019) regarding outcomes and causes of mortality on this species from two of the main wildlife rehabilitation centers in the north of Portugal. A total of 740 animals were admitted; the majority were juveniles, with the highest admission rate occurring during summer (36.8%). The main cause of admission was debilitation (30.7%). Of the total number of individuals admitted to these centers, 66.6% were released successfully. The main cause of death was trauma of unknown origin (32.7%).

**Abstract:**

This study aimed to analyze the admission causes, outcomes, primary causes of death, and main lesions observed in the post mortem examinations of Western European hedgehogs, *Erinaceus europaeus* (Linnaeus, 1758), in the north of Portugal. The data were obtained by consulting the records from the two main wildlife rehabilitation centers located in the north of Portugal (Wildlife Rehabilitation Centre of Parque Biologico de Gaia and the Wildlife Rehabilitation Centre of the University of Trás-os-Montes and Alto Douro). Over 17 years (2002–2019) a total of 740 animals were admitted. Most of the animals were juveniles, with the highest number of admissions occurring during summer (36.8%) and spring (33.2%). The main reasons for admission were debilitation (30.7%) and random finds (28.4%). Of the total number of individuals admitted to these centers, 66.6% were successfully released back into the wild. The most relevant causes of death were trauma of unknown origin (32.7%), nontrauma causes of unknown origin (26.6%), and nutritional disorders (20.2%). The main lesions observed were related to trauma, including skeletal and skin lesions (fractures, hemorrhages, wounds) and organ damage, particularly to the lungs and liver. The hedgehog is a highly resilient and adaptable animal. The urban environment has many benefits for hedgehogs, yet the presence of humans can be harmful. In the future, the public needs to become even more involved in the activities of the wildlife centres, which will make a positive difference for these populations.

## 1. Introduction

The Western European hedgehog, *Erinaceus europaeus* (Linnaeus, 1758), is a generalist nocturnal mammal, widely distributed across the European continent [1,2,3]. It hibernates from November to March, with some periods of awakening to forage or move in its nest [4]. Its diet consists mainly of macroinvertebrates, although, due to their great trophic adaptation potential, they can be generalist feeders [1,5]. This species has been classified as Least Concern (LC) in Portugal, according to the International Union for Conservation of Nature’s (IUCN) Red List of Threatened Animals [6]. Recent monitoring data from the Netherlands, Sweden, United Kingdom (UK), Belgium, and Germany show that its population has suffered a decline in recent decades [7,8]. Agricultural intensification, habitat fragmentation, road traffic accidents, molluscicide and rodenticide poisoning, and predation (e.g., by foxes, badgers, dogs) have been suggested amongst the major causes of this decline [5,7,8,9].

*E. europaeus* is one of the species that seem to prefer urban areas as their current habitat. These animals are commonly found on green spaces in constructed areas such as gardens and parks [4,7]. Some studies in the UK have suggested that the hedgehog decline is more severe in rural than in urban areas. Urban areas have become a suitable habitat for hedgehogs due to the higher food densities associated with human occupation, the existence of additional nest sites, and a decreased risk of predation by their natural predators [4,6,7,10]. 

Hedgehogs are one of the most common mammal species admitted to wildlife rehabilitation centers, sanctuaries, or veterinary hospitals. The main reasons for admission described in the literature include skin, respiratory and gastrointestinal diseases, malnutrition, hypothermia, and traumatic injuries [4,5]. Due to their preference to inhabit urban areas, hedgehogs are subjected to a higher risk of human-related traumatic injuries, which can include drowning, injuries inflicted by domestic pets, poisoning, entrapment, and road injuries [4,5,7]. 

Even though data on causes of mortality and morbidity of hedgehogs have been published previously, long-term studies about admissions of these animals to rehabilitation centers are quite scarce, particularly in southern Europe. 

The main purpose of this study was to collect data from hedgehog admittance records from the two major wildlife rehabilitation centers located in the north of Portugal, describing admission causes, outcomes, primary causes of death, and main lesions observed in the post mortem exams. 

## 2. Materials and Methods

This study was conducted at the Wildlife Rehabilitation Centre of Parque Biológico de Gaia (WRC-PBG) (41°05′48.50″ N–8°33′21.34″ W), which is located on the northern Portuguese coastline, and at the Wildlife Rehabilitation Centre of University of Trás-os-Montes and Alto Douro (CRAS-UTAD) (41°17′18.13″ N–7°44′21.94″ W) in inland northern Portuguese. 

For this study, we examined all hedgehog (*E. europaeus*) admittance records from these WRCs between 2002 and 2019. The variables analyzed were the arrival date, age (categorized as young or adult, based on their external characteristics), sex, the primary reason for admission, outcome, and post mortem examinations of the dead animals. 

For each animal, the reason for admission was determined as one of the following: random find (healthy animals that were found in gardens, roads, or inside buildings), orphaned, debilitated, held in captivity (animals that were kept in captivity by people as pets for a period of time), dead, or injured, according to previous studies [11,12,13].

The outcome of each animal was categorized as follows: euthanized (EU), died during the recovery process (DE), and released into the wild (RE). The cause of death was determined based on clinical signs and post mortem examination, including a histopathological exam. The cause of death was categorized as infectious and parasitic disease, nutritional disorder, poisoning, nontraumatic death of unknown origin, collision with vehicles, predation (both from natural predators and dog/cat attacks), neoplasia, trapping, or trauma of unknown origin.

The post mortem examinations were performed in line with the established technique and safety procedures for this species [14]. The macroscopic exams were performed by I.P., A.G., V.S., and S.L., and the histopathological exams by I.P. All the macroscopic findings were recorded in a written protocol and using photographs. For the histopathological examinations, representative samples were collected from each animal. Samples were fixed in buffered 10% formalin solution, embedded in paraffin wax, sectioned at 3 μm, and stained with HE (hematoxylin-eosin). 

All data collected were organized in Excel sheets, and the descriptive statistics, normality test, and inferential analyses were performed using SPSS Advanced Models TM 21.0 (SPSS Inc. 233 South Wacker Drive, 11th Floor Chicago, IL 60606-6412, USA). To study the differences between the observed and expected frequencies of categories of a field, one-sample nonparametric tests were used (binomial test or chi-square test, depending on the number of categories of the categorical field).

## 3. Results

### 3.1. Descriptive Data

**Figure 1:**
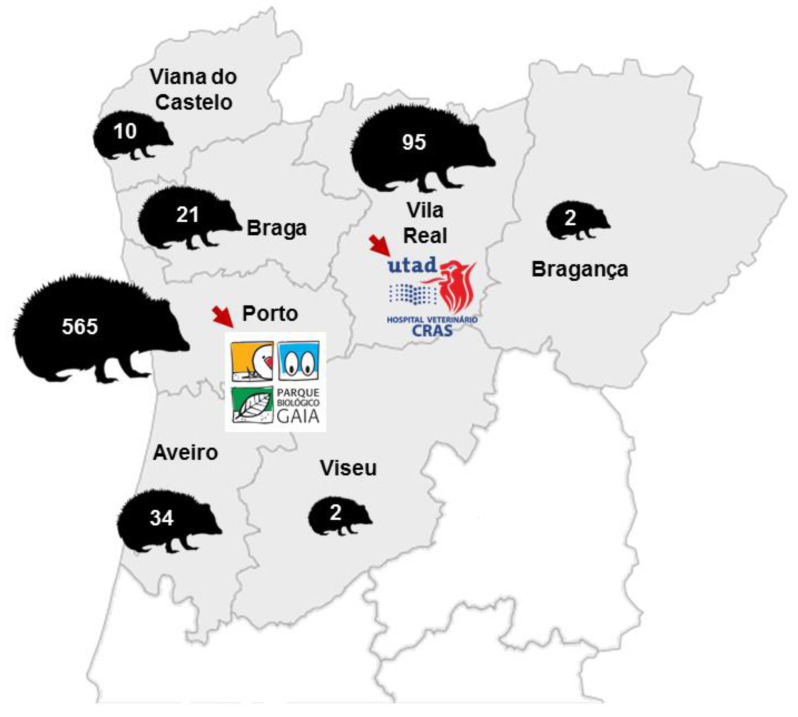
The number of animals distributed across different districts of origin in the north of Portugal, based on the locations from which they were collected. The locations of the Wildlife Rehabilitation Centre of Parque Biológico de Gaia (WRC-PBG) (41°05′48.50″ N–8°33′21.34″ W) and the Wildlife Rehabilitation Centre of University of Trás-os-Montes and Alto Douro (CRAS-UTAD) (41°17′18.13″ N–7°44′21.94″ W) are marked.

A total of 740 animals were admitted to the WRC-PBG (n = 636) and CRAS-UTAD (n = 104), between 2002 and 2019. Figure 1 shows a map with the distribution of the admitted animals by the different district of origin in northern Portugal, based on the location from which they were collected. The majority came from Porto (n = 565) and Vila Real (n = 95).

In only 90 out of the 740 animals was it possible to determine their sex, with 52% (n = 47) being females and 48% (n = 43) males. The differences observed between animal sexes were not significant (*p* = 0.706). 

Regarding the age, 155 animals were juveniles, 49 were adults, and in the remaining 536 age was not identified. The difference between adults and juveniles was statistically significant (*p* < 0.0001).

The season with the highest hedgehog admission rate was the summer (36.8%) followed by spring (33.2%), autumn (22.3%), and winter (7.6%). Statistically significant differences were observed between seasons (*p* < 0.001).

**Figure 2:**
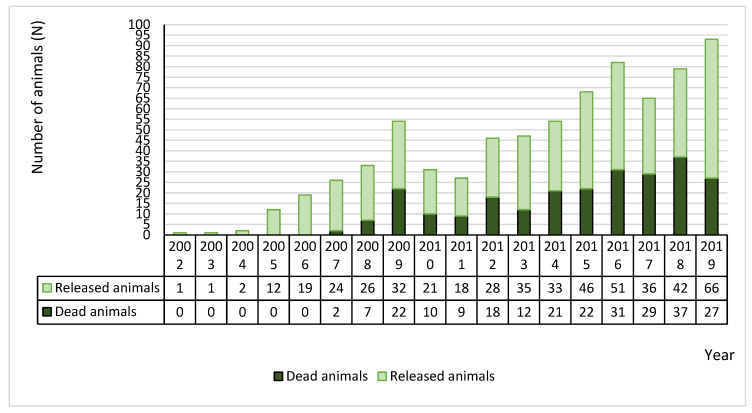
The number of animals admitted from 2002 to 2019, with the number of dead and released animals per year.

The animals admitted to both WRCs and later released back to the wild represented 66.6% (n = 492) of the cases, and 33.3% (n = 248) died. Figure 2 represents the total number of animals admitted from 2002 to 2019, along with the number of dead and released animals per year.

### 3.2. Reasons for Admission to the WRCs

**Figure 3:**
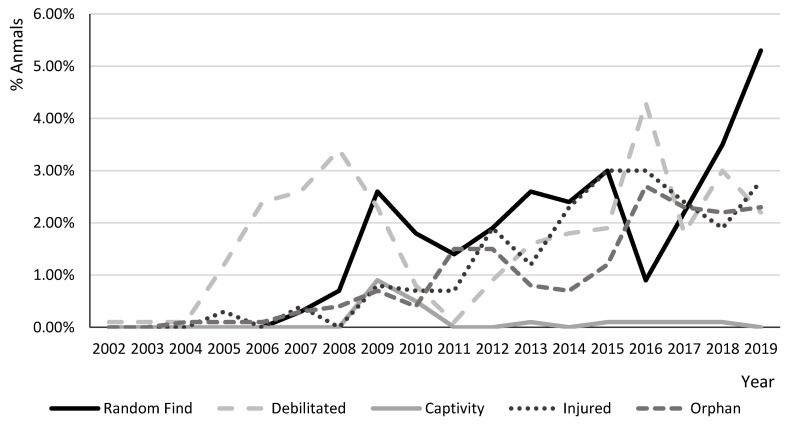
Distribution of the reasons for admission of *Erinaceus europaeus* to both wildlife rehabilitation centers from 2002 to 2019.

The main reasons for admission were as follows: 30.7% (227) debilitated, 28.4% (n = 211) random finds, 21.4% (n = 158) injured, 17.3% (128) orphaned, and 2.2% (n = 16) held in captivity. There was a significant difference between the reasons for admission (*p* < 0.01). (Figure 3, Table S1) represents the distribution, in percentages, of the reasons for admission over the different years. 

Table 1 displays the frequency and percentage of the reasons for admission by season, sex, age, and outcome. 

As shown in Table 1, the seasons with the highest number of admissions were summer and spring for the categories random find, orphan, and debilitated (*p* < 0.0001). The injured and held in captivity categories had slightly higher numbers of admissions during autumn. 

Regarding age and season, there were significant differences. During winter, most of the animals admitted were adults, while in the other seasons juveniles were the most common (*p* < 0.001). Although more females were admitted in spring and summer, and more males in winter and autumn, there were no significant differences (*p* = 0.55).

Except for injured animals, the predominant outcome across all categories was the release back to the wild. 

There were statistically significant associations between cause of admission and the seasons (*p* < 0.001); in the winter and spring, most animals were admitted due to a debilitating physical condition, in the autumn due to injuries, and in the summer due to random finding.

### 3.3. Causes of Death

Of the 248 animals that died, 83.5% (n = 207) died during treatment and 16.5% (n = 41) were euthanized. Of those animals 7.7% (n = 19) were females, 9.2% (n = 23) were males, and in the remaining 83.1% (n = 206) sex was not identified. Pertaining to the age, 8.1% (n = 20) were adults, 21.7% (n = 54) were juveniles, and in the remaining 70.2% (n = 174) the age was not determined.

The highest percentage of death occurred during summer (n = 92, 37.1%), followed by spring (n = 74, 29.8%), autumn (n = 64, 25.8%), and winter (n = 18, 7.3%) (Table 2).

**Figure 4:**
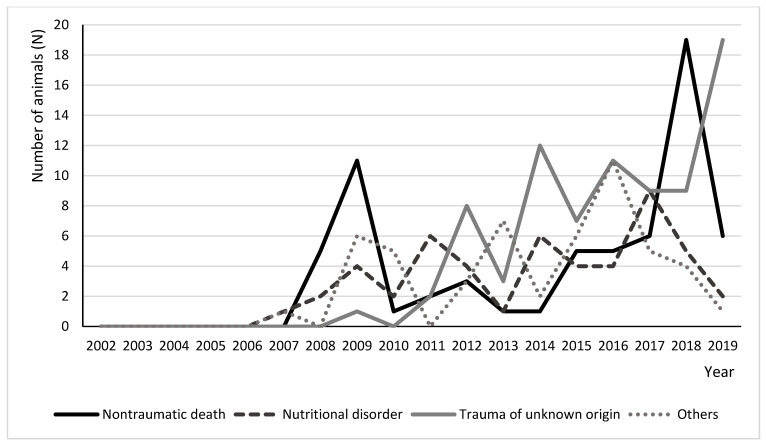
Distribution of the main causes of death of *Erinaceus europaeus* in both wildlife rehabilitation centers from 2002 to 2019. The category “others” includes predation, neoplasia, trapping, collision with vehicles, infectious and parasitic diseases, and poisoning.

In decreasing order of frequency, the causes of death were as follows: 32.7% (n = 81) trauma of unknown origin, 26.6% (n = 66) nontraumatic death of unknown origin, 20.2% (n = 50) nutritional disorder, 8.1% (n = 20) infectious or parasitic disease, 4.8% (n = 12) predation, 4.8% (n = 12) collision with vehicles, 1.6% (n = 4) poisoning, 0.8% (n = 2) trapping and 0.4% (n = 1) neoplasia. (Figure 4, Table S2) represents the distribution of the different causes of death from 2002 to 2019. Table 3 presents the frequency and percentage of the causes of death by the type of death and cause of admission.

### 3.4. Post Mortem Findings

Post mortem exams were performed on the 248 animals. Post mortem injuries were observed in different organs and systems. This section describes the main macroscopic lesions observed, complemented with some microscopic findings. 

**Figure 5:**
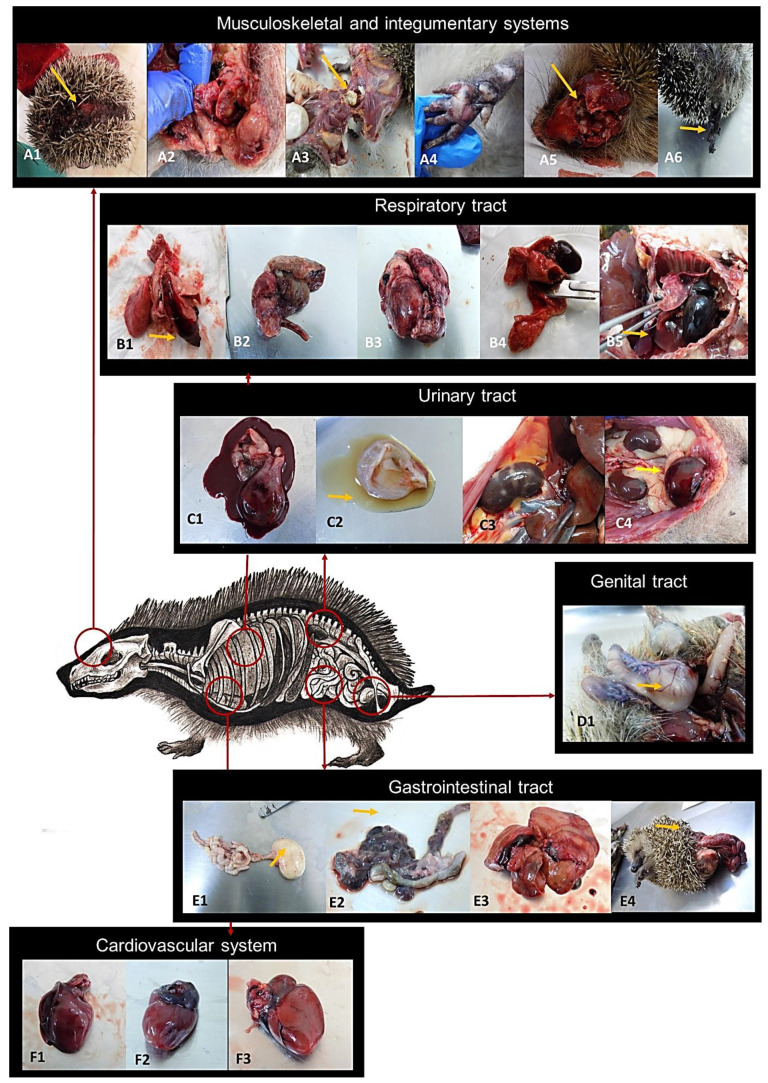
Schematic representation of lesion observed during the post mortem exams of *Erinaceus europaeus*. **A1**—skin wound; **A2**—pelvis fracture; **A3**—cervical abscess; **A4**—pododermatitis; **A5**—skull fracture; **A6**—posterior limb amputation; **B1**—lung hemorrhage; **B2**—fibrinous bronchopneumonia; **B3**—emphysema; **B4**—parasitic pneumonia; **B5**—hemothorax; **C1**—hemorrhagic cystitis; **C2**—altered urine pigmentation; **C3**, **C4**—kidney congestion; **D1**—pyometra; **E1**—gastric dilatation; **E2**—enteritis; **E3**—hepatic fracture and lipidosis; **E4**—evisceration; **F1**—myocardial discoloration; **F2**, **F3**—myocardial congestion.

Upon external examination, 170 animals revealed signs of dehydration and emaciation. In all animals, the presence of ectoparasites such as fleas, mites (the most common being *Caparinia tripilis*), and ticks (mainly *Ixodes hexagonus*) was registered to various degrees. Three animals presented a high degree of parasitism, which led to emaciation, dehydration, and anemia and death. One animal presented a fungal infection due to dermatophytes (Figure 5). 

One hundred and seven animals had skeletal and skin lesions (Figure 5A1–A6). They presented mild to severe traumatic injuries: skin lacerations (50), evisceration (2) (Figure 5E4), spine fractures (6) tibial fracture (4), fracture of the femur (2), sacrum fracture (6), limb amputation (1) (Figure 5A6), pododermatitis (2), and fracture of the skull (3) (Figure 5A5). Twelve animals presented polytrauma associated with a vehicle collision. Twelve animals had a cerebral concussion lesion. At least five animals presented eye evisceration prolapse or enucleation and ear hemorrhage. Among the muscular lesions, we found pallid muscles, hemorrhagic muscles, and subcutaneous hematomas associated with the fractured bones. In one animal, an abscess was observed in a cervical vertebra (Figure 5A3), causing an abnormal angulation of the neck (45°) in the caudal direction and causing severe respiratory distress.

At the level of internal organs, several animals presented congestion (115) and had free sanguineous liquid in the thoracic (47 hemothorax) (Figure 5B5) and abdominal (80 hemoperitoneum) cavities associated with traumatic lesions. One animal presented ascites and several ruptures of the diaphragm (7). The majority of these animals had been run over by vehicles. 

Concerning the respiratory system, the lesion most commonly observed was congestion (50), followed by hemorrhage (44), edema (10), hydrothorax (1), and emphysema (2). Additionally, histological examination revealed bronchopneumonia (4), fibrinous bronchopneumonia (2), emphysematous areas of unknown etiology (3), interstitial pneumonia (3), aspiration pneumonia (15), hydrothorax (1), parasitic pneumonia (25), bronchitis (3), and emphysema (2) (Figure 5B1–B4). One animal presented a mucoid exudate in the nose and eyes. 

In the cardiovascular system, observations included alterations in the shape of the heart (1), myocardial discoloration (2), hemopericardium (6), and myocardial hemorrhage (9) (Figure 5F1–F3). Histological examination of areas of myocardial discoloration also revealed vacuolization in isolated myocytes (1). 

In the gastrointestinal tract, observations included split teeth (13), gingivitis, small wounds in the oropharynx and esophagus, hemorrhage (40), gastric ulcers (5), parasites (40), tympany (7) (Figure 5E1), enteritis of unknown origin (12) (Figure 5E2), hemorrhagic enteritis (2), intestinal rupture (5), and rectal prolapse (1). In the liver, nonspecific congestion (47), hepatic fractures (55), pale-discoloration lipidosis (116), subcapsular hemorrhages (1), and focal necrosis (5) (Figure 5E3) were observed. Additionally, in the histological exams, eosinophilic hepatitis, lipidosis, focal necrosis and hepatocellular atrophy were revealed (Figure 5). 

Splenic fractures (43), splenomegaly (5), and, upon histological examination, the presence of megakaryocytes (2) and granulomatous splenitis (1) were detected in the spleen. 

Two animals presented an enlargement of the adrenal gland with cortical hyperplasia.

In the urinary system, the main organ affected was the kidney, with nonspecific congestion and hemorrhage or circulatory lesions associated with trauma (97) (Figure 5C2–C4). Hemorrhagic cystitis (1) (Figure 5C1) and bladder rupture (3) were also observed. The histological examination exposed one focal non-purulent nephritis (1). 

In the genital tract, two females presented lesions, one with a closed pyometra (Figure 5D) and other with neoplasia in the mammary gland.

In some individuals (n = 17), it was not possible to investigate any lesions of the internal organs because the carcasses were too autolytic. The majority of the animals’ histopathological examinations were impaired, mostly by severe autolysis, and some were not performed due to lack of financial resources.

## 4. Discussion

The present study was the first investigation on outcomes, reasons for admission, mortality and pathological findings on wild hedgehogs in Portugal. Studies concerning wild hedgehogs’ mortality are very scarce and almost nonexistent. To our knowledge, within the Mediterranean region, there was a similar study performed by Martinez et al. (2014) [5] in Spain. In most cases, information regarding outcomes or mortality is dispersed in the grey bibliography such as annual reports of the wildlife rehabilitation centers [11,12] or related to a specific cause of death such as collisions with road vehicles [10,13,14]. 

Our data were drawn from the records of a large number of cases admitted to two wildlife rehabilitation centers, WRC-PBG and CRAS-UTAD, over 17 years (2002–2019). These institutions are the main centers that receive and treat wild terrestrial vertebrates on the north of the country. These centers directly depend on the national government branch that regulates the wildlife services, ICNF. They are equipped with adequate facilities to accommodate wildlife and a team of technicians and veterinarians that rehabilitate the animals (Portaria n. ° 1112/2009, 2009). The injured animals are received from different official organizations (Serviço de Proteção da Natureza e do ambiente (SEPNA), police, etc.) and directly from the general public. Both possess a large database that registers the maximum possible information about the admissions. 

A total of 740 hedgehogs were admitted during this period, with most of the animals being collected in the district of Porto and Vila Real. This was expected since these are the WRCs’ locations, and there is therefore a higher probability of their receiving injured and debilitated animals from the neighboring area; this was also noticed in similar studies concerning other species in this region [15,16].

There was no significant difference between the sexes of the animals admitted, in contrast to what was perceived in other studies [1,2,3]. Regarding age, most of the animals were juveniles. The main seasons of admission were summer (36.8%) and spring (33.2%). Similar data were observed in other studies, and are expected based on the animals’ natural behavior [4,6,7]. In spring, they emerge from the hibernation period and are more dispersed on the search of food. During the months of February–March and again in August–September, the males become more active during the reproductive season (in Portugal they can reproduce two times a year) [4,5]. In the months July–August (summer), females are more actively feeding and enlarging their home ranges after weaning their young, and the first juveniles start to disperse at the same time [4,6,7].

The main reasons for admission to the WRCs were debilitation, random finds, and injury. Admissions due to random finds and injury have been increasing in recent years. A similar trend has been observed in Spain [3] and the United Kingdom [2]. Nonetheless, in Spain, the main reasons for admission were random finds (40.8%) and orphaned young (19.4%). This difference could be related to many factors, such as differences in the habitat, human population, urbanization density, and how aware the public is of how to respond to an accidental find of wildlife. 

Regarding the outcomes of the animals admitted to the two WRCs, 66.6% were released back to the wild, following the results described by Martinez et al. (2014) [5], with 69% of the admitted hedgehogs returned to the wild. Indeed, most of the admissions corresponded to healthy animals found accidentally or animals kept for a short period in the WRC when they were slightly underweight, dehydrated, or the weather was too cold [1]. Hedgehogs are resilient animals, which are relatively easy to keep in captivity, and recover well [5]. Most studies indicate that these animals, when released back in the wild, adapt and survive well [1,3,5].

Of the 248 dead animals, the vast majority, 83.4%, died during treatment. Several studies have suggested that there is no significant sex- or age-related specific mortality [1], and the same was observed in this study. The greatest peaks of mortality were in summer and spring, for the same reasons as explained previously. The main three causes of death were trauma of unknown origin (32.7%), nontraumatic death of unknown origin (26.6%), and nutritional disorder (20.2%). Unfortunately, one of the most common limitations of retrospective studies is the incomplete data. A large percentage of animals in this study presented unidentified cause of death, sex, or age. This could be related to the lack of information taken on the admittance form, scarce human and financial resources to perform complete examinations, and the absence of a digital system in which to store the data in the early years. The causes of death observed were oriented towards those described in the bibliography, where the main causes of death were associated with trauma, malnutrition, and dehydration [5,17,18,19]. The latter was present almost exclusively in juveniles, and is related to inexperience and early loss of their parents for diverse reasons [5,20]. Mortality in different years and regions can be related to variations in the surrounding environment. Some studies have shown that rigorous winters lead to greater mortality of individuals; this phenomenon has been observed in countries located in northern Europe such as Sweden, Denmark, Finland, and the United Kingdom [7,8,17,18]. Similarly, in urban areas, after hibernation, animals can become more susceptible to accidental finds by humans and pets (traps, predation, collision, and others) when foraging. The availability of resources can be lower and more disputed in certain areas, such as those that are more industrialized [21,22,23]. As a result of the curious nature of hedgehogs, it seems that they are somewhat accident-prone and death through misadventure is not uncommon. It is common to observe hedgehogs drowning in garden ponds, entangled in netting, falling into holes (particularly cattle grids), or licking poisonous substances [20]. Since hedgehogs seem to enjoy living in our gardens [3,18], it is common to observe these accidents. In our study, it was possible to identify some examples. Furthermore, direct contact with humans leads to a higher level of death related to anthropogenic sources, as traps, roadkill, and poisons, as was observed. 

The pathological findings confirmed the main causes of mortality of these animals to be associated with trauma. The main lesions observed were related to trauma, with skeletal and skin lesions (fractures, hemorrhages, open wounds) and organ damage, particularly of the lungs and the liver. In this study, there was probably a greater number of animals with parasitic and infectious disease, but, due to the lack of complementary exams, it was not possible to confirm all diagnoses. In one case it was possible to identify the first case of a pyometra described in this species in a wild individual [24]. 

From 2002 to 2019, it was possible to observe an increase in the number of hedgehog admissions to the WRCs. This phenomenon could be related to the increase in the general public interest in wildlife and greater knowledge of the daily work of the WRCs [25]. An important part of the WRCs’ mission is to educate the general public (particular youngsters) regarding wild fauna and flora, being a powerful tool for environmental awareness [26,27]. In terms of hedgehogs, the WRCs are making an effort to generate greater involvement of members of the public in the rehabilitation process of these animals. Due to the animal’s peculiar aspect, small size, and friendly behavior, it is often used in environmental campaigns as a symbol, allowing the public, mostly children, to get involved in its recovery. These activities have great conservation value by allowing the public to understand more about WRCs’ daily work and projects, acquiring knowledge about natural history, ecology, and threats on these wild species, particularly those severely affected by anthropogenic factors [1,4,28]. They also help to provide adequate information to people living in peri-urban and residential areas, allowing them to identify situations when rescue is necessary and to deliver animals to WRCs whenever special care is needed [4,5,18].

## 5. Conclusions

In conclusion, of the 740 animals admitted to two wildlife rehabilitation centers in the north of Portugal, more than half recovered and were released back to the wild. The main causes of admission were related to random finds and debilitating physical conditions. In this region, the main causes of death of these animals were associated with trauma, and in most cases linked to anthropogenic sources. The hedgehog is a highly resilient and adaptable animal. This is the first time that such a long study related to outcomes and mortality has been performed for this species. The urban environment has benefits for hedgehogs, offering supplementary sources of food and shelter, but the presence of humans may harm them. It is important that in the future, the public can become even more involved in the activities of wildlife centers and similar environmental associations, allowing them to make a positive difference to hedgehog populations.

## Figures and Tables

**Table 1:** Frequency and percentage of the reasons for admission by season, sex, age, and outcome.

	Random Find	Debilitated	Captivity	Injured	Orphaned
**Season**					
Winter	12 (21.4)	33 (58.8)	2 (3.6)	6 (10.7)	3 (5.4)
Autumn	41 (24.8)	44 (26.7)	6 (3.6)	52 (31.5)	22 (13.3)
Spring	68 (27.6)	75 (30.5)	5 (2.0)	50 (20.30)	48 (19.50)
Summer	90 (33.0)	75 (27.6)	3 (1.1)	50 (18.4)	55 (20.2)
**Sex**					
Female	20 (42.6)	7 (14.9)	1 (2.1)	11 (23.4)	8 (17.0)
Male	21 (48.8)	4 (9.30)	1 (2.3)	8 (16.8)	9 (20.9)
**Age**					
Adult	31 (63.3)	8 (16.3)	2 (4.1)	8(16.3)	0
Juvenile	22 (14.2)	3 (1.9)	0	8(5.2)	122(78.7)
**Outcome**					
Death	30 (14.3)	76 (33.0)	2 (12.5)	105 (65.8)	35 (27.4)
Released	180 (85.7)	152 (67.0)	14 (87.5)	54 (34.2)	92 (71.9)

**Table 2:** Frequency and percentage of the causes of death by season, sex, and age.

	Nontraumatic Death					Trauma			
	Nutritional Disorders	Poisoning	Infectious and Parasitic Diseases	Neoplasia	Nontraumatic Death of Unknown Origin	Predation	Trapping	Collision with Vehicles	Trauma of Unknown Origin
**Season**									
Winter	0	0	7 (38.8)	1 (5.5)	6 (33.3)	1 (5.5)	0	3 (16.6)	0
Autumn	6 (9.3)	1 (1.6)	4 (6.2)	0	14 (21.8)	5 (7.9)	1 (1.6)	2 (3.1)	31 (48.4)
Spring	20 (27.0)	1 (1.4)	3 (4.1)	0	21 (28.4)	4 (5.4)	1 (1.4)	4 (5.4)	20 (27.0)
Summer	24 (26.1)	2 (2.2)	6 (6.5)	0	25 (27.2)	2 (2.2)	0	3 (3.2)	30 (32.6)
**Sex**									
Female	6 (31.6)	2 (10.5)	4 (21.1)	1 (5.2)	1 (5.2)	2 (10.5)	0	2 (10.5)	1 (5.2)
Male	6 (26.1)	1 (4.3)	6 (26.1)	0	3 (13.0)	1 (4.3)	1 (4.3)	2 (8.7)	3 (13.0)
**Age**									
Adult	3 (15.0)	2 (10.0)	8 (40.0)	1 (5.0)	1 (5.0)	1 (5.0)	0	3 (15.0)	1 (5.0)
Juvenile	44 (81.4)	1 (1.8)	4 (7.4)	0	1 (1.8)	2 (3.7)	0	1 (1.8)	1 (1.8)

**Table 3:** Frequency and percentage of the causes of death by the type of death and reason for admission (euthanized—EU, died during the recovery process—DE).

	Nontraumatic Death					Trauma			
	Nutritional Disorders	Poisoning	Infectious and Parasitic Diseases	Neoplasia	Nontraumatic Death of Unknown Origin	Predation	Trapping	Collision with Vehicles	Trauma of Unknown Origin
**Type of death**									
DE	45 (21.7)	3 (1.4)	17 (8.2)	1 (0.5)	62 (30.0)	6 (2.8)	2 (1.0)	10 (4.8)	61 (29.5)
EU	5 (12.2)	1 (2.4)	3 (7.3)	0	4 (9.7)	6 (14.6)	0	2 (4.9)	20 (48.8)
**Reason for admission**									
Random find	10 (33.3)	1 (3.3)	5 (16.6)	1 (3.3)	8 (26.6)	0	0	4 (13.3)	1 (3.3)
Debilitated	4 (5.3)	2 (2.6)	12 (16.0)	0	55 (73.3)	0	0	1 (1.3)	1 (1.3)
Captivity	0	0	0	0	2 (100)	0	0	0	0
Injured	1 (0.9)	1 (0.9)	2 (1.9)	0	1 (0.9)	12 (11.5)	2 (1.9)	7 (6.7)	79 (75.2)
Orphan	35 (97.2)	0	1 (2.7)	0	0	0	0	0	0

## Supplementary Materials

The following are available online at https://www.mdpi.com/2076-2615/10/8/1305/s1, Table S1: Distribution of the reasons for admission of *Erinaceus europaeus* in both wildlife rehabilitation centers from 2002 to 2019, Table S2: Distribution of the causes of death of *Erinaceus europaeus* in both wildlife rehabilitation centers from 2002 to 2019.
