# Supplementary material for: Outcomes, Mortality Causes, and Pathological Findings in European Hedgehogs (Erinaceus europeus, Linnaeus 1758): A Seventeen Year Retrospective Analysis in the North of Portugal
Source: Animals (Basel). 2020 Jul 30;10(8):1305. doi: 10.3390/ani10081305 (PMC7460247; doi:10.3390/ani10081305)
Supplement: Supplementary file 1 [file animals-10-01305-s001.pdf]

Supplementary Materials

Table S1. Distribution of the causes of admission of *Erinaceus europaeus* in both Wildlife Rehabilitation Centers from 2002 to 2019.

Year	Random Find	Debilitated	Captivity	Injured	Orphan
2002	0.00%	0.10%	0.00%	0.00%	0.00%
2003	0.00%	0.10%	0.00%	0.00%	0.00%
2004	0.00%	0.10%	0.00%	0.00%	0.10%
2005	0.00%	1.20%	0.00%	0.30%	0.10%
2006	0.00%	2.40%	0.00%	0.00%	0.10%
2007	0.30%	2.60%	0.00%	0.40%	0.30%
2008	0.70%	3.40%	0.00%	0.00%	0.40%
2009	2.60%	2.30%	0.90%	0.80%	0.70%
2010	1.80%	0.80%	0.50%	0.70%	0.40%
2011	1.40%	0.10%	0.00%	0.70%	1.50%
2012	1.90%	0.90%	0.00%	1.90%	1.50%
2013	2.60%	1.60%	0.10%	1.20%	0.80%
2014	2.40%	1.80%	0.00%	2.30%	0.70%
2015	3.00%	1.90%	0.10%	3.00%	1.20%
2016	0.90%	4.30%	0.10%	3.00%	2.70%
2017	2.20%	1.80%	0.10%	2.40%	2.30%
2018	3.50%	3.00%	0.10%	1.90%	2.20%
2019	5.30%	2.20%	0.00%	2.80%	2.30%

Table S2. Distribution of the causes of death of *Erinaceus europaeus* in both Wildlife Rehabilitation Centers from 2002 to 2019.

Year	Trapping	Collision with vehicles	Poisoning	Infectious and parasitic diseases	Non-trauma of unknown origin	Neoplasia	Nutritional disorders	Predation	Trauma of unknown origin
2002	0	0	0	0	0	0	0	0	0
2003	0	0	0	0	0	0	0	0	0
2004	0	0	0	0	0	0	0	0	0
2005	0	0	0	0	0	0	0	0	0
2006	0	0	0	0	0	0	0	0	0
2007	0	0	0	0	0	0	1	1	0
2008	0	0	0	0	5	0	2	0	0

2009	0	3	0	2	11	0	4	1	1
2010	0	2	0	1	1	0	2	2	0
2011	0	0	0	0	2	0	6	0	2
2012	0	1	0	0	3	1	4	1	8
2013	0	0	1	5	1	0	1	1	3
2014	0	0	0	2	1	0	6	0	12
2015	1	0	2	2	5	0	4	1	7
2016	0	3	0	6	5	0	4	2	11
2017	1	0	0	2	6	0	9	2	9
2018	0	1	1	1	19	0	5	1	9
2019	0	1	0	0	6	0	2	0	19
